# The application of multiple metrics in deformable image registration for target volume delineation of breast tumor bed

**DOI:** 10.1002/acm2.13793

**Published:** 2022-10-20

**Authors:** Xin Xie, Yuchun Song, Feng Ye, Hui Yan, Shulian Wang, Xinming Zhao, Jianrong Dai

**Affiliations:** ^1^ Department of Radiation Oncology, National Cancer Center/National Clinical Research Center for Cancer/Cancer Hospital Chinese Academy of Medical Sciences and Peking Union Medical College Beijing China; ^2^ Department of Diagnostic Radiology, National Cancer Center/National Clinical Research Center for Cancer/Cancer Hospital Chinese Academy of Medical Sciences and Peking Union Medical College Beijing China

**Keywords:** deformable image registration, postoperative radiotherapy, target volume delineation, tumor bed

## Abstract

**Background and purpose:**

For postoperative breast cancer patients, deformable image registration (DIR) is challenged due to the large deformations and non‐correspondence caused by tumor resection and clip insertion. To deal with it, three metrics (fiducial‐, region‐, and intensity‐based) were jointly used in DIR algorithm for improved accuracy.

**Materials and methods:**

Three types of metrics were combined to form a single‐objective function in DIR algorithm. Fiducial‐based metric was used to minimize the distance between the corresponding point sets of two images. Region‐based metric was used to improve the overlap between the corresponding areas of two images. Intensity‐based metric was used to maximize the correlation between the corresponding voxel intensities of two images. The two CT images, one before surgery and the other after surgery, were acquired from the same patient in the same radiotherapy treatment position. Twenty patients who underwent breast‐conserving surgery and postoperative radiotherapy were enrolled in this study.

**Results:**

For target registration error, the difference between the proposed and the conventional registration methods was statistically significant for soft tissue (2.06 vs. 7.82, *p* = 0.00024 < 0.05) and body boundary (3.70 vs. 6.93, *p* = 0.021 < 0.05). For visual assessment, the proposed method achieved better matching result for soft tissue and body boundary.

**Conclusions:**

Comparing to the conventional method, the registration accuracy of the proposed method was significantly improved. This method provided a feasible way for target volume delineation of tumor bed in postoperative radiotherapy of breast cancer patients.

## 1 INTRODUCTION

Breast cancer has become the most frequently diagnosed cancer type. It has also caused many cancer deaths in women all over the world.^1^ Breast‐conserving surgery, followed by supportive radiotherapy, has become the established treatment of early‐stage breast cancer.^2^ For postoperative breast cancer radiotherapy, it is crucial to accurately delineate the tumor bed and its target volume. The target volume delineation, however, is susceptible to the number of surgical clips, clarity and size of seroma, interobserver variability, specimen volume, and other factors.^3^ Moreover, because of asymmetric excision of the tumor, the uniform expansion of the resection cavity does not well represent clinical target volume (boost‐CTV) to some extent.^4^

Registering preoperative and postoperative images and calculating the tumor contour propagation can help target volume delineation in radiotherapy. However, the image registration is difficult for breast given the nature of soft tissue.^5^, ^6^ Breast could undergo complex and large deformation as a nonrigid structure. It is difficult to align the preoperative and postoperative CT images through rigid registration due to breast surgery and postsurgical changes.^7^, ^8^, ^9^ Recently, deformable image registration (DIR) has been shown to add value in defining the breast tumor bed and its target volume using various modalities, including contrast CT,^10^ CT,^11^, ^12^, ^13^ MRI,^14^ and PET‐CT.^15^ Overall, comparing to rigid registration methods, DIR methods could achieve higher registration accuracy.

For registering preoperative and postoperative images, most DIR methods of breast in radiotherapy are intensity‐based methods. However, the image intensities of tumor region and tumor bed are quite different because of tumor resection, clip insertion, and postsurgical changes. Moreover, the accuracy of intensity‐based DIR (i‐DIR) method will be compromised by these intensity changes to a certain degree.^16^, ^17^ Given the fact that the registration accuracy is closely related to the accuracy of target volume delineation, it is of necessity to improve the registration accuracy. For geometric features, such as anatomic landmarks and contours of region of interests (ROIs), studies have shown that incorporating them with intensity‐based methods can achieve higher registration accuracy.^18^, ^19^ Recently, the hybrid method that combines anatomical landmarks with image intensities has proven to be effective.^20^ However, there are no reports on the application of contour information. The ROIs in breast that could be used to guide image deformation are almost unavailable (except for unaffected breast gland), which precludes the use of contour to some extent.

So far, several commercial registration software, such as Velocity,^15^ MIM,^21^ and Mirada,^10^ have been employed by researchers. The performances of these software in contour propagation have been fully investigated.^22^ Although very little overlap was found for the target volumes in Yu's research,^14^ most studies have demonstrated the value of DIR in contour delineation. However, the DIR methods they used in commercial registration software were essentially intensity‐based, which were not appropriate for the problem. As for the hybrid method that combined anatomical landmarks with image intensities, the researcher merely evaluated registration accuracy, without an assessment of clinical contour. For more comprehensive evaluation, Wodzinski et al. assessed different state‐of‐the‐art i‐DIR methods.^23^, ^24^ Furthermore, artificial deformations, which model the tumor bed creation process, were proposed to validate the deformation correctness.^23^ However, the studies focused mainly on tumor bed but not boost‐CTV. It was worth noting that boost‐CTV was defined as the expansion of tumor bed and involved more surrounding soft tissue. Thus for DIR methods, especially in the case when image intensities showed large differences, more attention could be paid to the soft tissue alignment rather than local discontinuity near the tumor bed.

In this study, in addition to intensity‐based metric, fiducial‐based and region‐based metrics were introduced as constraints in the DIR method for higher registration accuracy. In Section 2, the principle of the multi‐metric registration was explained in detail. Then, the evaluation methods, including target registration error (TRE), contour propagation, and visual assessment, were introduced. Next, the performances of the proposed method were summarized in Section 3. Finally, the advantages and disadvantages of the proposed method were discussed, and the future work was prospected.

## 2 MATERIALS AND METHODS

### 2.1 Datasets

Twenty early‐stage breast cancer patients who underwent breast‐conserving surgery (BCS) and eligible for whole breast irradiation (WBI) plus boost irradiation were enrolled in this study. Fifteen patients had left‐sided breast cancer, and the other five had right‐sided breast cancer. The median age of patients was 58 years (range, 44–67 years), and the pathological diagnosis was all invasive ductal carcinoma with a stage of T1‐T2N0M0. No patient received oncoplastic surgery. All patients underwent a lumpectomy with sentinel lymph node dissection, and tumor‐negative margins were ensured during a single operation. Equal or more than five surgical clips were used to mark the boundaries of the lumpectomy cavity. All enrolled patients had either no seroma or a seroma clarity score of ≤3 in the lumpectomy cavity. None of the patients had chronic lung disease, and all exhibited normal arm movement after surgery. This study was approved by the local Ethics Committee, and written informed consent was obtained from all enrolled patients.

The image data consisted of 20 preoperative CT images and 20 planning CT images, which were both non‐contrast‐enhanced. The preoperative CT image was acquired averagely 1 day before surgery, whereas the planning CT image was acquired averagely 10 weeks after surgery. The two sets of CT images were acquired in the same radiotherapy treatment position, where the patients were in the supine position, immobilized on a breast bracket with no degree of incline, and placed using arm support (with both arms above the head). The CT images were scanned using a Somatom Definition AS 40 (Siemens Healthcare, Forchheim, Germany) or a Brilliance CT Big Bore (Philips Healthcare, Best, the Netherlands) and were reconstructed using a matrix of 512 × 512, a slice thickness of 5.0 mm, and a pixel size of 0.68–1.37 mm.

All contours were delineated by the same radiation oncologist on both the preoperative CT and the planning CT images using the Pinnacle treatment planning system (Phillips Medical Systems). On the planning CT images, the tumor bed was contoured according to the surgical clips, seroma, and postoperative changes, and boost‐CTV was expanded from the tumor bed with a 1‐cm margin three‐dimensionally and limited to 5 mm from the skin surface. PTV was obtained by adding a 5‐mm margin in all directions to the CTV and was limited to 5 mm from the skin surface. All tumors were identifiable on the preoperative CT images and delineated by the same radiation oncologist. Moreover, tumor+1, tumor+2, and tumor+3 were obtained by adding a 1‐, 2‐, 3‐cm margin three‐dimensionally to the tumor region and was limited within the skin surface. The volumes used for contour propagation analysis are listed in Table 1. The volume of tissue resected (pathological volume) was measured by the maximum diameter in three dimensions. Moreover, the calculated volume was 57.58 ± 52.13 cm^3^ (mean ± standard deviation), which was most approximate to the volume of tumor+2.

**TABLE 1:** The volumes (cm^3^) used for contour propagation analysis

Volumes	Mean ± SD
Tumor	1.61 ± 1.20
Tumor+1	21.44 ± 7.60
Tumor+2	59.64 ± 20.13
Tumor+3	107.80 ± 36.42
Tumor bed	14.26 ± 9.37
Boost‐CTV	60.50 ± 25.72

Abbreviations: CTV, clinical target volume; SD, standard deviation.

### 2.2 Multi‐metric registration

**FIGURE 1:**
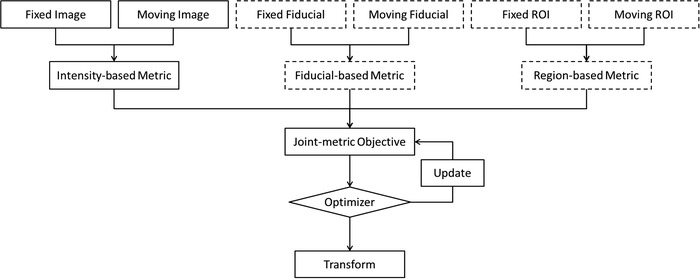
Flowchart of the multi‐metric registration process

The flowchart of the multi‐metric registration process is illustrated in Figure 1. Dashed boxes represent the new components (fiducial‐based metric and region‐based metric), whereas solid boxes represent the conventional intensity‐based registration procedure.

Fiducial‐based DIR method minimizes the distance between two paired fiducial point sets with known correspondence. It is defined as the mean Euclidean distance^25^

(1) $$S_{CP}=\frac{1}{P}\;\sum_{x_{F_{i}}}∥x_{M_{i}}-T_{\mu }\left(x_{F_{i}}\right)∥$$

where *P* is the number of paired points, and $x_{F_{i}}$ and $x_{M_{i}}$ are a pair of fiducial points in the fixed image (planning CT) and the moving image (preoperative CT), respectively. In a patient average 30 paired points located in unaffected breast gland were automatically marked through Harris Corner Detection method (function provided in 3D slicer) and confirmed by the radiation oncologist.

Region‐based DIR method utilizes Kappa statistic (KS) to maximize the overlap of paired ROIs. KS (1994_kappa origin) is defined as^26^

(2) $$\mathrm{KS}\;=\frac{2\times \left|A\&B\right|}{\left(\left|A\right|+\left|B\right|\right)}$$

where *A* is the foreground region in the moving image, *B* is the foreground region in the fixed image, & is intersection, and |.| indicates the area of the enclosed set. In the computation of the metric, only foreground pixels (binary segments) are considered. In this study, the unaffected breast gland was contoured using automatic gray‐level thresholding segmentation method (function provided in 3D slicer) and confirmed by the radiation oncologist.

i‐DIR method maximizes mutual information (MI) between image intensities, which is defined as^27^

(3) $$\begin{array}{ccc} & & \text{MI}\left(\mu ;I_{F},I_{M}\right)\\ & & =\sum_{m\varepsilon L_{M}}\;\sum_{f\in L_{F}}p\left(f,m;\mu \right)\log_{2}\left(\frac{p\left(f,m;\mu \right)}{p_{F}\left(f\right)p_{M}\left(m;\mu \right)}\right) \end{array}$$

where $L_{F}$ and $L_{M}$ are sets of regularly spaced intensity bin centers, *p* is the discrete joint probability, and $p_{F}$ and $p_{M}$ are the marginal discrete probabilities of the fixed and moving image, obtained by summing *p* over *m* and *f*, respectively.

The multi‐metric objective is defined as

(4) $$C\;\left(T_{\mu };I_{F},I_{M}\right)=\frac{1}{\sum_{i=1}^{N}\omega_{i}}\;\sum_{i\;=\;1}^{N}\omega_{i}C_{i}\left(T_{\mu };I_{F},I_{M}\right)$$

with $w_{i}$ is the weight of $C_{i}$ metric, which is one of fiducial‐based (1), region‐based (2), and intensity‐based (3) metrics. This way multiple metrics can jointly be optimized during a registration.

The publicly available open‐source software, 3D Slicer (version 4.11.0),^28^ was used for data management and display. The i‐DIR toolkit, Elastix,^29^, ^30^ was employed for voxel‐to‐voxel image registration through the SlicerElastix extension. The affine transformation was first used for initial alignment. Then, a multi‐resolution registration scheme was used for B‐Splines transform. The Gaussian pyramid (3 scales) was adopted to down‐sample and smooth the image at different scales. The grid size of control points was set to 12. The grid space of each scale was 4, 2, and 1 mm. The larger grid size was used for matching larger structures and skipping smaller structures, whereas the smaller grid size was specified for matching detailed structures. Stochastic gradient descent optimization was used with up to 500 iterations at each multi‐resolution level. The optimal weights of fiducial‐, region‐, and intensity‐based metrics were decided as 1, 1, and 100, respectively, through trial and error.^31^ The PC equipped with Intel Core i5 CPU 3.4 GHz and 32 GB RAM was used to perform all the registration tasks.

### 2.3 Evaluations

The proposed method was compared with the conventional intensity‐based registration method using several criteria, including TRE, contour propagation, and visual assessment. Moreover, contour propagation performances were quantified using geometric criteria, the Dice similarity coefficient (DSC), and the Hausdorff distance (HD), between the reference target volume and the deformed tumor contours.

**FIGURE 2:**
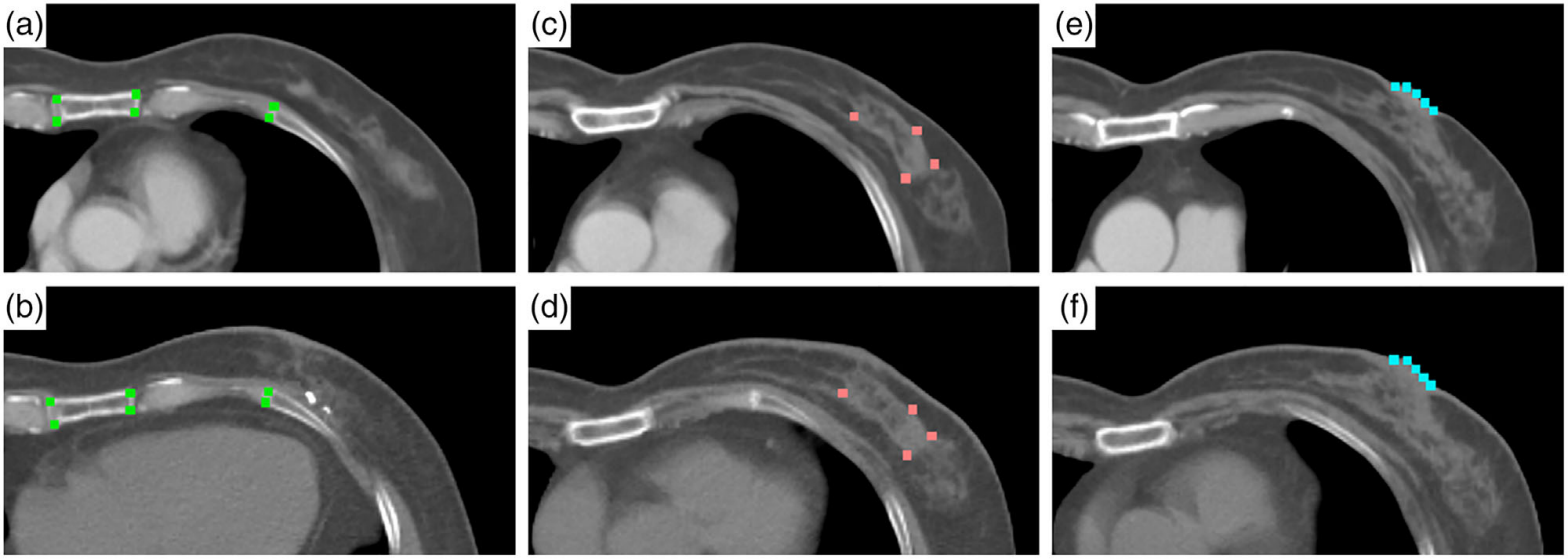
Three groups of points selected for calculating target registration error (TRE). These points are represented by dots with different colors (green: rigid structures; pink: soft tissues; blue: body boundaries): (a) rigid structures in moving image; (b) rigid structures in fixed image; (c) unaffected soft tissues in moving image; (d) unaffected soft tissues in fixed image; (e) body boundaries in moving image; and (f) body boundaries in fixed image

TRE refers to Euclidean distance between corresponding points in the transformed moving image and the fixed image. Mean TRE is as follows^32^:

(5) $$\mathrm{TR}E_{\mathrm{mean}}=\frac{1}{I}\;\sum_{i}\sqrt{{\left(x_{i}-{x_{i}}^{\prime }\right)}^{2}+{\left(y_{i}-{y_{i}}^{\prime }\right)}^{2}+{\left(z_{i}-{z_{i}}^{\prime }\right)}^{2}}$$

where *i* is single point indicator, *I* is total number of points, and (*x*,*y*,*z*) and (*x*′,*y*′,*z*′) are the coordinates of point in the fixed and the transformed moving image. As shown in Figure 2, each patient had 30 pairs of points based on their locations in three types of tissue: rigid structures (ribs, breastbone), unaffected soft tissues (unaffected breast gland within 5 cm from tumor), and body boundaries (nipple). It is worth noting that the points located in unaffected soft tissue can be more reliable than the other two groups because they are the closest to tumor region. Specifically, the points located in unaffected soft tissues were automatically marked through Harris Corner Detection method (function provided in 3D slicer) and confirmed by the radiation oncologist. Moreover, these points used in the TRE analysis were different from the ones used for the fiducial‐based objective.

Contour propagation performances were quantified using geometric criteria between the reference target volume and the deformed tumor contours. Delineated tumor+2 and tumor+3 were deformed separately for different DIR methods, in order to avoid potential uncertainties caused by the deformation of multiple contours.^33^ The spatial overlaps between deformed tumor+2 and tumor bed, deformed tumor+3 and boost‐CTV were calculated using the DSC and the HD.

The DSC is defined as follows^34^:

(6) $$\mathrm{DSC}\;\left(A,B\right)=\frac{2\left|A\cap B\right|}{\left|A\right|+\left|B\right|}$$

where *A* is the reference target volume manually delineated by the radiation oncologist; *B* is the tumor contour deformed by the calculated transformations (and propagated onto the planning CT image); *A* ∩ *B* is the volume that *A* and *B* have in common. The DSC results in values between 0 and 1, where 0 represents no intersection and 1 reflects perfect overlap.

The HD is defined as^35^

(7) $$\mathrm{HD}\;\left(A,B\right)=\;\max\left(h\left(A,B\right),h\left(B,A\right)\right)$$

where

(8) $$h\left(A,B\right)=\max_{a\in A}\left(\min_{b\in B}\left\|a-b\right\|\right)$$

and ∥·∥ is some underlying norm on the points of *A* and *B* (e.g., the *L*_2_ or Euclidean norm). h(A,B) identifies the point *a* ∈ *A* that is farthest from any point of *B* and measures the distance from a to its nearest neighbor in *B*. The HD(A,B) is the maximum of h(A,B) and h(B,A) and measures the largest degree of mismatch between *A* and *B*. The overlap between *A* and *B* increases with smaller HD(A,B).

Visual assessments, including checkerboard image and image overlay display, were used to check the quality of registration results based on the experience of radiation oncologist. It is particularly effective for checkerboard image to identify mismatches at tissue interface of high contrast.^36^ Besides, the blended composition of registered images is presented in image overlay display. Color orange and cyan represent the fixed and the transformed moving image, respectively. Moreover, perfect alignment is displayed in gray. Each patient's CT images were evaluated by two experienced radiation oncologists within 3–4 min.

For testing purpose, the conventional i‐DIR method was performed as standard result. Moreover, three combinations of multi‐metric DIR methods were investigated, including region‐ and i‐DIR (ri‐DIR) method, fiducial‐ and i‐DIR (fi‐DIR) method, and fiducial‐, region‐, and i‐DIR (fri‐DIR) method. For statistical analysis, the paired *t*‐test was performed if the data were normally distributed. Otherwise, the Wilcoxon signed‐rank test for paired samples (nonparametric test) was performed. A level of *p* < 0.05 was considered statistically significant. All statistical analyses were performed in R statistical software (version 3.6.3) (https://www.r‐project.org/).

## 3 RESULTS

**FIGURE 3:**
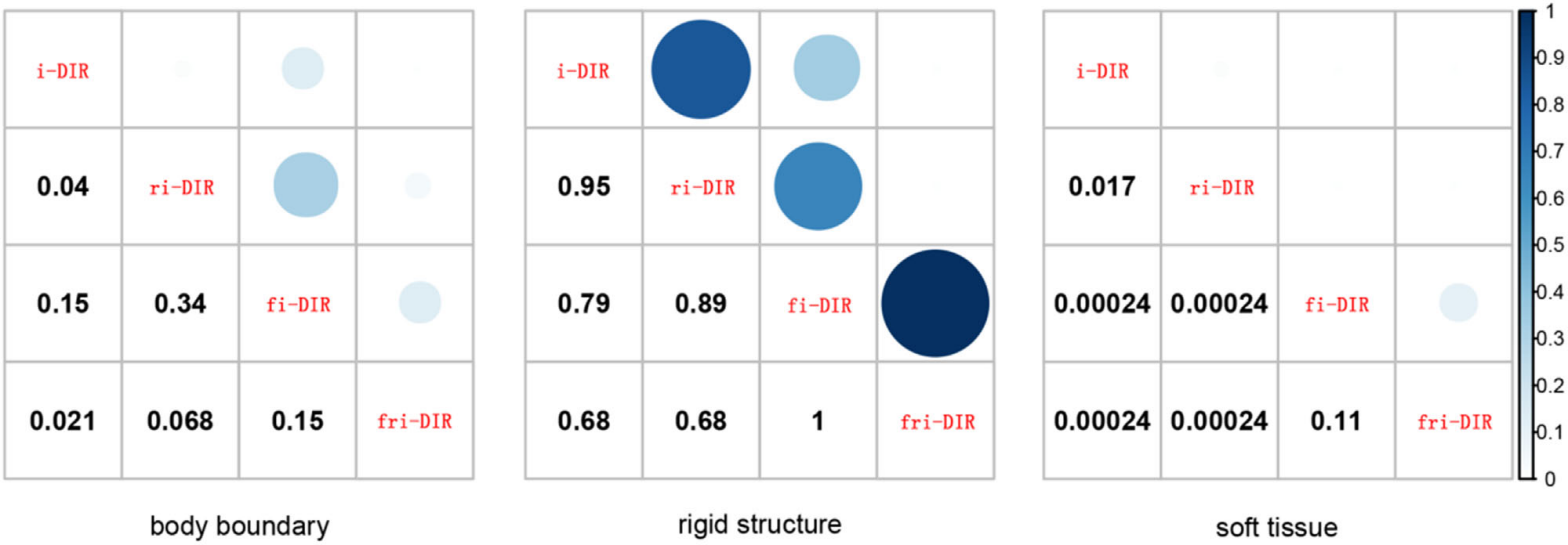
The *p*‐values for comparisons of the four deformable image registration (DIR) methods in target registration error (TRE) results. A level of *p* < 0.05 was considered statistically significant.

The statistics of TRE for the four DIR methods are listed in Table 2. Moreover, the *p*‐values for comparisons are shown in Figure 3. The Shapiro–Wilk normality test results confirmed that the data were not normally distributed, so the Wilcoxon signed‐rank test was performed. The conventional i‐DIR method achieved mean TREs of 7.82, 3.34, and 6.93 mm for soft tissue, rigid structure, and boundaries, respectively. For soft tissue and boundary alignment, the fri‐DIR method improved the registration accuracy to the best. The fi‐DIR method showed higher accuracy than the ri‐DIR method. Compared with the i‐DIR method, the *p*‐values were all less than 0.05, which indicated significance with the introduction of fiducial‐based and region‐based metrics. Except for boundary alignment, there was no statistical difference between the i‐DIR and the fi‐DIR methods. Specifically, the *p*‐value was 0.15. In evaluating rigid structures, although statistically insignificant, a slight decrease in registration error was observed between the i‐DIR method and three multi‐metric methods.

**TABLE 2:** The results (mean ± standard deviation) of target registration error (TRE; mm) corresponding to three groups of point sets for the four deformable image registration (DIR) methods

Methods	Soft tissues	Rigid structures	Body boundaries
i‐DIR	7.82 ± 4.91	3.34 ± 2.52	6.93 ± 6.00
ri‐DIR	5.80 ± 4.00	3.31 ± 3.49	5.30 ± 4.37
fi‐DIR	2.17 ± 0.52	3.10 ± 3.20	3.97 ± 2.19
fri‐DIR	2.06 ± 0.39	3.02 ± 2.82	3.70 ± 2.18

Abbreviations: fi‐DIR, fiducial‐ and intensity‐based deformable image registration; fri‐DIR, fiducial‐, region‐, and intensity‐based deformable image registration; i‐DIR, intensity‐based deformable image registration; ri‐DIR, region‐ and intensity‐based deformable image registration.

The results of contour propagation for the four DIR methods are listed in Table 3. Moreover, the *p*‐values for comparisons are shown in Table 4. The results of Shapiro–Wilk normality test confirmed that the data were normally distributed, so the paired *t*‐test was used. For DSC, the fi‐DIR and the fri‐DIR method both improved the overlap between the reference target volume and the deformed tumor contours (deformed tumor+2 and tumor bed, which deformed tumor+3 and boost‐CTV) to the best. Compared with the i‐DIR method, the differences were statistically significant (*p* = 0.0025, 0.0047 < 0.05). For HD, the fri‐DIR method improved the overlap to the best. Overall, although statistically insignificant, a slight improvement of overlap was observed between the i‐DIR method and three multi‐metric methods.

**TABLE 3:** The Dice similarity coefficient (DSC) and the Hausdorff distance (HD) results (mean ± standard deviation) of contour propagation (HD; mm) for the four deformable image registration (DIR) methods

Methods	(Deformed tumor+2)–(tumor bed)		(Deformed tumor+3)–(boost‐CTV)	
	DSC	HD	DSC	HD
i‐DIR	0.29 ± 0.12	7.68 ± 1.92	0.55 ± 0.11	6.24 ± 2.00
ri‐DIR	0.32 ± 0.14	7.20 ± 2.01	0.57 ± 0.12	5.76 ± 1.88
fi‐DIR	0.33 ± 0.15	7.01 ± 1.95	0.58 ± 0.12	5.67 ± 1.80
fri‐DIR	0.33 ± 0.15	6.99 ± 1.98	0.58 ± 0.12	5.61 ± 1.78

Abbreviations: CTV, clinical target volume; fi‐DIR, fiducial‐ and intensity‐based deformable image registration; fri‐DIR, fiducial‐, region‐, and intensity‐based deformable image registration; i‐DIR, intensity‐based deformable image registration; ri‐DIR, region‐ and intensity‐based deformable image registration.

**TABLE 4:** The *p*‐values for comparisons of the four deformable image registration (DIR) methods in contour propagation results

Comparisons	(Deformed tumor+2)–(tumor bed)		(Deformed tumor+3)–(boost‐CTV)	
	DSC	HD	DSC	HD
i‐DIR vs. ri‐DIR	0.095	0.081	0.15	0.087
i‐DIR vs. fi‐DIR	0.0025 (**)	0.07	0.19	0.17
i‐DIR vs. fri‐DIR	0.0047 (**)	0.086	0.24	0.14
ri‐DIR vs. fi‐DIR	0.061	0.23	0.52	0.66
ri‐DIR vs. fri‐DIR	0.077	0.23	0.63	0.46
fi‐DIR vs. fri‐DIR	0.47	0.74	0.65	0.28

Abbreviations: CTV, clinical target volume; DSC, Dice similarity coefficient; fi‐DIR, fiducial‐ and intensity‐based deformable image registration; fri‐DIR, fiducial‐, region‐, and intensity‐based deformable image registration; HD, Hausdorff distance; i‐DIR, intensity‐based deformable image registration; ri‐DIR, region‐ and intensity‐based deformable image registration.

^**^ stands for a level of p < 0.01, which is considered statistically significant.

**FIGURE 4:**
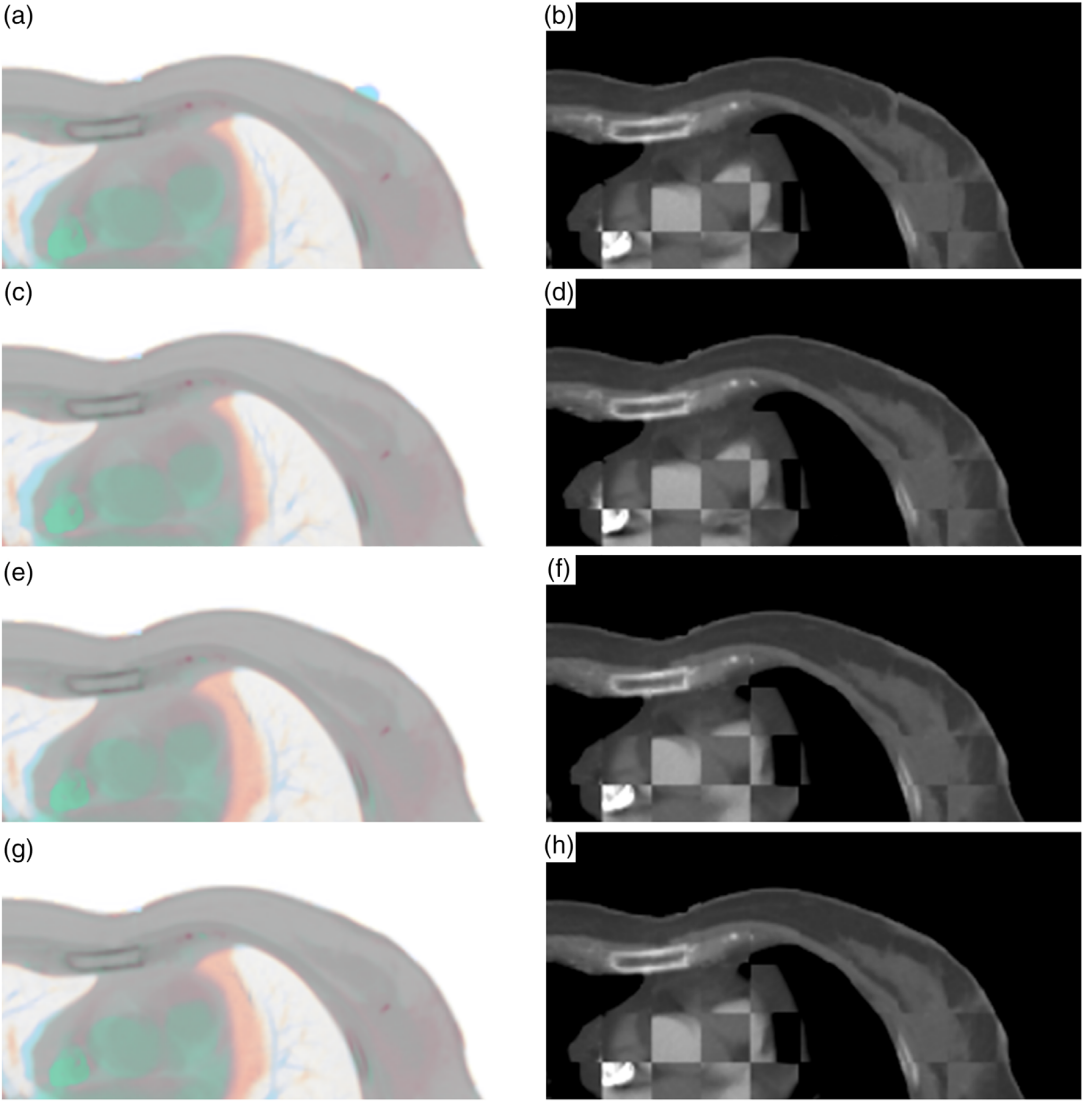
Image overlay displays and checkerboard images for visual assessment: (a) image overlay display of intensity‐based deformable image registration (i‐DIR); (b) checkerboard image of i‐DIR; (c) image overlay display of region‐ and intensity‐based deformable image registration (ri‐DIR); (d) checkerboard image of ri‐DIR; (e) image overlay display of fiducial‐ and intensity‐based deformable image registration (fi‐DIR); (f) checkerboard image of fi‐DIR; (g) image overlay display of fiducial‐, region‐, and intensity‐based deformable image registration (fri‐DIR); and (h) checkerboard image of fri‐DIR

For qualitative visual assessment, the image overlay displays and the checkerboard images between the fixed and the transformed moving images are shown in Figure 4. Visual assessment of three multi‐metric methods was better than that of the i‐DIR method, which was consistent with the TRE's evaluation. Among three multi‐metric methods, the fri‐DIR method achieved optimal matching for soft tissue and boundary. Moreover, fi‐DIR method performed better than ri‐DIR method.

## 4 DISCUSSION

In this study, anatomic landmarks and contours of ROIs were incorporated with i‐DIR method for higher registration accuracy. Application of geometric features in breast DIR was an attempt in the field of postoperative breast cancer radiotherapy and showed great potential for clinical use. Based on the results, the hybrid method proposed was less affected by the large intensities changes. Moreover, the introduction of KS for ROIs had the ability to restrict unrealistic image deformation and keep the DIR result more reasonable.

For TRE results, compared with the i‐DIR method, the fi‐DIR method showed greater improvement than the ri‐DIR method as shown in Table 2. Furthermore, although the fri‐DIR method improved the registration accuracy to the best, it did not have a very distinct advantage over the fi‐DIR method. Thus from the results, the fiducial‐based metric would be more reliable than the region‐based metric. The improvement of the ri‐DIR method is limited, which may be attributed to the fact that the contours of unaffected breast gland were not accurate enough.

In this study, tumor+2 on preoperative CT image was assumed to be close to the volume of tissue resected (based on the calculated volume shown in Table 1). Thus for contour propagation, the deformed tumor+2 and tumor+3 were regarded as the tumor bed and boost‐CTV, respectively. However, the uniform expansion of tumor (tumor+2) is not necessarily close to the real volume of tissue resected due to the asymmetric excision of the tumor in clinical practice. As shown in Table 3, although the improvement of overlap was observed between the i‐DIR method and three multi‐metric methods, the DSC results were still far less than the recommended tolerance of 0.8–0.9.^36^ This may be partly due to the fact that the accuracy of ground truth tumor bed/boost‐CTV is affected by many factors. Besides, more precise range of resected volume on preoperative CT image should be investigated. The tumor contour propagated onto the planning CT using DIR could lead to a more accurate modification of RT contours to a certain degree.

Qualitative visualization of the registration results is subjective. However, it should not be ignored because it is the easiest way to eliminate inappropriate deformation from the clinical point of view. In the study, the region near the tumor bed can be more reliable in assessing accuracy, so it is proper to inspect unaffected soft tissues more carefully. It is worth noting that the differences between the three multi‐metric methods were not apparent. Thus, TRE is needed in addition to the qualitative visual assessment.

There are several limitations of this study. First, the image datasets are limited. More data could be collected in the future. Second, breast tumors might not be easily identified from surrounding tissues on CT images, especially for those without contrast agent usage. Given the poor contrast of tumor and normal tissues on CT images, additional image modalities (such as MRI), which have superior contrast, could be considered. Third, the traditional automatic methods for point detection (Harris Corner Detection) and contour segmentation (gray‐level thresholding segmentation) were less accurate, especially in low contrast regions. This procedure can potentially be improved by other novel methods or deep learning methods in terms of precision and efficiency.^37^, ^38^, ^39^ Fourth, the slice resolution of CT image in this study was limited to 5 mm due to clinical protocol. A finer thickness (<3 mm) will result in better registration accuracy, where the improvement is marginal with 1 mm according to previous reports.^40^ Therefore, 3 mm is preferred for clinical use and will be adopted in future study. Last, this study focused merely on WBI plus boost irradiation. However, accelerated partial breast irradiation has become an alternative to WBI for patients with low risk of recurrence.^41^ Moreover, for patients receiving oncoplastic surgery, the delineation of the tumor bed and boost volume would be much more complicated. The proposed method could be investigated for these patients in the future.

## 5 CONCLUSIONS

The introduction of fiducial‐ and region‐based metrics into the conventional i‐DIR method was effective to register pre‐ and postoperative CT images for patients undergone BCS and proceeded to radiotherapy. The influence of large deformations and non‐correspondence on registration accuracy was reduced with the proposed method. This method provided a feasible way for target volume delineation of tumor bed in postoperative breast cancer radiotherapy.

## AUTHOR CONTRIBUTIONS

Xin Xie performed the experiments, analyzed the data, and wrote the manuscript. Yuchun Song and Feng Ye collected the data and delineated the target volumes. Hui Yan contributed to the data analyses and manuscript preparation. Shulian Wang and Xinming Zhao contributed to the interpretation of the results and supervision of data collection. Jianrong Dai supervised the whole study. All authors have read and approved the manuscript.

## CONFLICT OF INTEREST

The authors have no relevant conflicts of interest to disclose.

## Data Availability

The data that support the findings of this study are available from the corresponding author upon reasonable request.
